# Helmet–Head Decoupling in Ice Hockey Impacts: An In-lab Exploratory Study Using Autoregressive Modeling of Acceleration Data Measured from a Helmet-Mounted Inertial Measurement Unit (IMU)

**DOI:** 10.1007/s10439-025-03848-2

**Published:** 2025-09-25

**Authors:** Dario Sciacca, Anisoara Ionescu

**Affiliations:** Signal Processing Laboratory 5, Swiss Federal School of Technology (EPFL), 1015 Lausanne, Switzerland

**Keywords:** Head impact biomechanics, Instrumented ice hockey helmets, Helmet–head decoupling, Inertial Measurement Unit (IMU), Concussion

## Abstract

**Purpose:**

This study aims to develop and validate in-lab a novel approach for estimating head linear acceleration in ice hockey impacts using IMU-instrumented helmets. The use of AutoRegressive (AR) modeling was investigated as a solution to mitigate the decoupling observed between the helmet and the head.

**Methods:**

A series of impacts were conducted on a helmeted Hybrid III 50th percentile male Anthropometric Test Device (ATD). The impacts were performed using a custom-built pendulum impactor in four directions (front, front–oblique, side and back–oblique) and at two energies, 33 and 79 J, except for the back–oblique direction, which was tested only at 33 J. The processing pipeline included impact segmentation, main direction estimation and application of the AR-based transfer function modeling. The error with respect to the reference signals from the headform was quantified and the transformed signals were compared with the unprocessed (raw) and lowpass filtered signals. The generalization capabilities of the transfer function were also evaluated on a different helmet type.

**Results:**

The application of the transfer function resulted in a reduction of up to 9.04 g (57%) and 27.54% for the average Root Mean Squared Error (RMSE) and peak Mean Absolute Percentage Error (MAPE), respectively, with a consistent error decrease across all impact directions, compared to the lowpass filtered signal. However, when evaluated on a different helmet model, the transfer function showed larger errors.

**Conclusion:**

The proposed methodology effectively improves the estimation of head linear acceleration across all impact directions. Nevertheless, performance varies with helmet type, indicating the need for helmet-specific adjustments (e.g., through model retraining).

**Supplementary Information:**

The online version contains supplementary material available at 10.1007/s10439-025-03848-2.

## Introduction

Concussions in contact sports have received increased attention due to their high occurrence rates and the growing understanding of their potential long-term effects. The mechanics of concussion are commonly accepted to involve either a direct impact to the head, face, neck or an impact elsewhere on the body that transmits a sudden force to the head [1–4]. Various hypotheses have been proposed regarding injury mechanisms, including linear motions (i.e., head translation), which can create pressure gradients within the skull and cause focal injuries [5–7] and angular motions (i.e., head rotations), which generate shear stresses and tensile strains that may result in diffuse axonal injuries [8–10]. While rotational accelerations and velocities are usually considered the primary contributors to injury [11, 12], it is suggested that both linear and rotational motions should be considered for a comprehensive assessment [5, 10, 13]. To date, the majority of research has focused on developing new kinematics and model-estimated response variables, known as Brain Injury Criteria, and evaluating their correlation with head injury [14]. One of the main challenges lies in the fact that these indices rely on the kinematics at the head Center of Mass, CoM [14], which cannot be measured in vivo.

Consequently, several methods have been proposed to measure head impacts in contact sports, all aiming to relate to the actual motions at the brain’s CoM. The use of instrumented helmets, equipped with accelerometers or a combination of accelerometers and angular rate sensors, have been investigated to assess head impacts, mainly in football and, to some extent, in ice hockey [15]. However, the relative head–helmet movement occurring during an impact can lead to inaccurate measurements of linear and rotational accelerations [5]. In response to this, instrumented mouthguards have emerged as a promising solution, since located in a strategic position that closely follows the head CoM motion [16–19]. On the other hand, mouthguards also present challenges for professional players, particularly regarding fit and comfort [20]. Therefore, finding a balance between accurate measurement and practical usability is nowadays a critical challenge for head impact assessment.

Anthropometric Test Devices (ATDs) are used to measure the kinematics at the CoM of a headform designed to simulate human morphology. These ATDs are typically instrumented with high performance accelerometers and gyroscopes, allowing researchers to compare the performance of helmet-mounted sensors against the headform as a reference. Joodaki et al. [21] investigated the head–helmet relative movement during football impacts using a helmeted ATD in combination with an optical motion capture system. Their results showed that the maximum resultant linear acceleration experienced by the helmet was approximately 2 to 5 times greater than that of the head. Additionally, the maximum resultant angular velocity of the helmet varied from 37% lower to 71% higher than that of the head, depending on the impact conditions. Kent et al. [22] conducted a study to characterize the helmet-to-ground effects in football, revealing that during ground impacts, the rebound of the head caused a 41% greater change in angular velocity at the head's CoM compared to the helmet. Allison et al. [23] investigated the effect of sensor location on a helmet, using a triaxial accelerometer and a triaxial gyroscope (GForceTracker, GForceTracker, Inc., Canada). The helmeted ATD was impacted at different intensities and directions, revealing average percentage differences in raw peak resultant linear acceleration greater than 100% between helmet and head. The raw peak resultant rotational velocity ranged between 10 and 15%. Results also showed that sensor placement affected linear acceleration measurements. Positioning sensors internally on the superior part of the helmet, closer to the head CoM reduced errors by up to 23.7%, compared to the other locations. These discrepancies highlighted the need for an appropriate transfer function that relates helmet sensors data to the head kinematics.

Such a transfer function has been proposed for the Head Impact Telemetry or HIT (Simbex, United States) system, which consists of multiple single-axis accelerometers arranged in an orthogonal configuration with respect to the head surface. The transfer function developed by Crisco et al. [24] is integrated to project the impact signal from the helmet sensors to the head CoM. Allison et al. [25] conducted a study to validate the HIT system against an instrumented Hybrid III 50th percentile male head model for ice-hockey head impacts. Average errors in peak acceleration between the HIT and headform varied from 18 to 31% and from 35 to 64% for linear and rotational acceleration, respectively. Rowson et al. [26] evaluated the HIT system integrated into a football helmet during head impacts using a pneumatic linear impactor on a Hybrid III head. Average relative errors with respect to the head for peak linear and rotational acceleration were 1% ± 18% and 3% ± 24%, respectively. The Root Mean Squared Error (RMSE) over the 25 ms temporal response was 12.5 ± 8.32 g for linear acceleration and 907 ± 685 rad/s^2^ for rotational acceleration. The same helmet (Riddell Evolution, Riddell, United States) was tested with two different sizes by Jadischke et al. [27], using a linear impactor on a Hybrid III head. Impacts with an absolute percentage error greater than 15% constituted 55% and 74% of the total impacts for linear and rotational acceleration, respectively.

This study aims to develop and validate in-lab a novel approach to estimate head kinematics using IMU-equipped ice hockey helmets. This methodology introduces the application of AutoRegressive (AR) modeling in the context of head impacts. An autoregressive model makes predictions on the variable of interest using a linear combination of its past values [28]. AR models have been used for different applications, ranging from environmental and economic forecasting [29, 30] to assessment and diagnostics in the medical field [31–34]. This is the first study to explore the feasibility of AR modeling specifically for developing helmet-to-head transfer function in ice hockey.

## Materials and Methods

### Measurement Setup

**Fig. 1:**
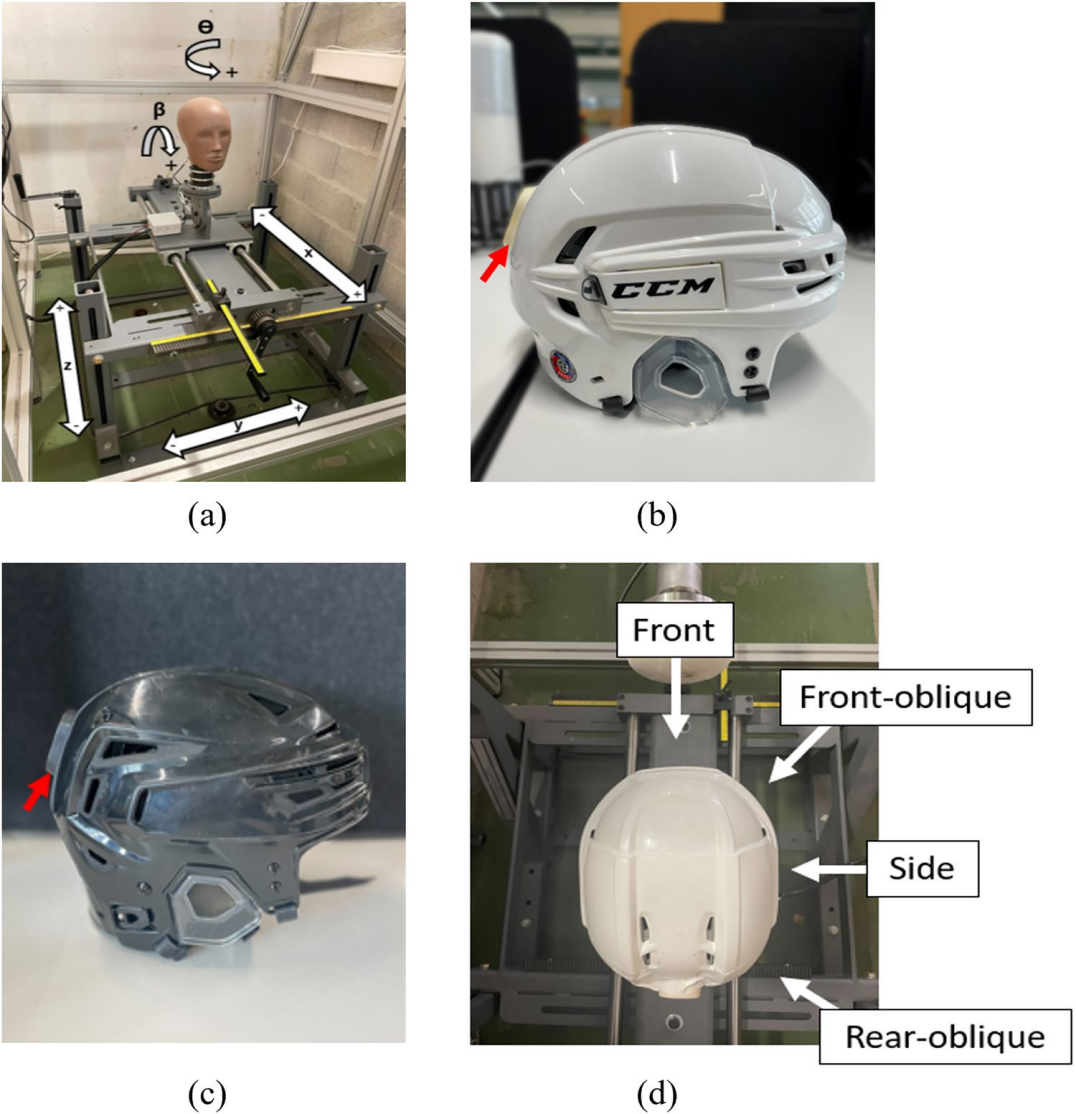
**a** Biokinetics slider table and carriage, **b** IMU-instrumented CCM Tacks 910 helmet, and **c** IMU-instrumented Bauer RE-AKT 150 helmet. The red arrows indicate the case containing the IMU; **d** impact directions

A series of impacts were executed on a certified head and neck Hybrid III 50th percentile male (Diversified Technical Systems DTS, United States) ATD mounted onto a sliding carriage (Biokinetics and Associates Ltd., Canada), using a custom-built pendulum impactor (see Sect. 1 in Supplementary Material). Pendulum impactors are recommended for their increased repeatability and reproducibility compared to other methodologies [35]. The sliding carriage allows the initial position and rotation of the head to be adjustable to 5 degrees of freedom, as shown in Fig. 1a. A data acquisition system comprised of three uni-axial linear accelerometers (Endevco 7264C-2000, Diversified Technical Systems DTS, United States, range ± 500 g) and three uni-axial angular rate sensors (ARS 8000, Diversified Technical Systems DTS, United States, range ± 8000 deg/s) was located at the ATD head center of mass. Two ice hockey helmets (Tacks 910, size M, CCM, Canada and RE-AKT 150, size M, Bauer, Canada) were instrumented with an IMU sensor (Bearmind SA, Switzerland), consisting of a triaxial accelerometer (ADXL375, Analog Devices, United States, range ± 200 g) and gyroscope (LSM6DSOX, STMicroelectronics, Switzerland, range: ± 2000 deg/s), with a total weight of approximately 12.53 g (including the associated protective case). The IMU was then inserted into a custom-designed case, which was attached using a two-sided adhesive tape (3M, United States) to the back of the helmets (Fig. 1b, c).

The helmets were placed on the headform in accordance with the manufacturers’ specifications. The ATD was impacted in the front, front–oblique, side and back–oblique directions (Fig. 1d). Starting from the front position (Fig. 1a, θ = 0, β = 0), each consecutive direction was obtained by applying a rotation θ of + 45° (right region) or − 45° (left region). To prevent potential damage to the helmet's IMU, no impacts were conducted on the back of the helmet. Two impact energies, 33 and 79 J, were achieved with the pendulum arm angle set to 35° and 50°, respectively. A digital inclinometer (Laserliner, Germany) was placed on the arm to measure the inclination. These energies correspond to impact velocities of approximately 3 and 4.6 m/s, respectively, which are among the velocities typically associated with sub-concussive or concussive head impacts [35]. For the back–oblique location, near the helmet IMU, only the lowest energy level was considered, in order to minimize the risk of compromising the sensor during the test. Fourteen trials were conducted for each impact configuration, defined by location and intensity, except for the back–oblique direction (15 trials in this case). Each trial consisted of three impacts with an in-between time of approximately 20 s. The impact tests were conducted by holding the impact energy constant while varying the impact location (i.e. all locations were tested sequentially at 33 J before proceeding to the next energy level, and so forth). The final test matrix is illustrated in the Table 1.

**Table 1:** Test matrix showing the total number of impacts for each location–intensity configuration

Location	33 J	79 J	Total
Front	42	42	84
Front–oblique	42	42	84
Side	42	42	84
Rear–oblique	45	0	45

### Data Analysis

#### Pre-processing

**Fig. 2:**
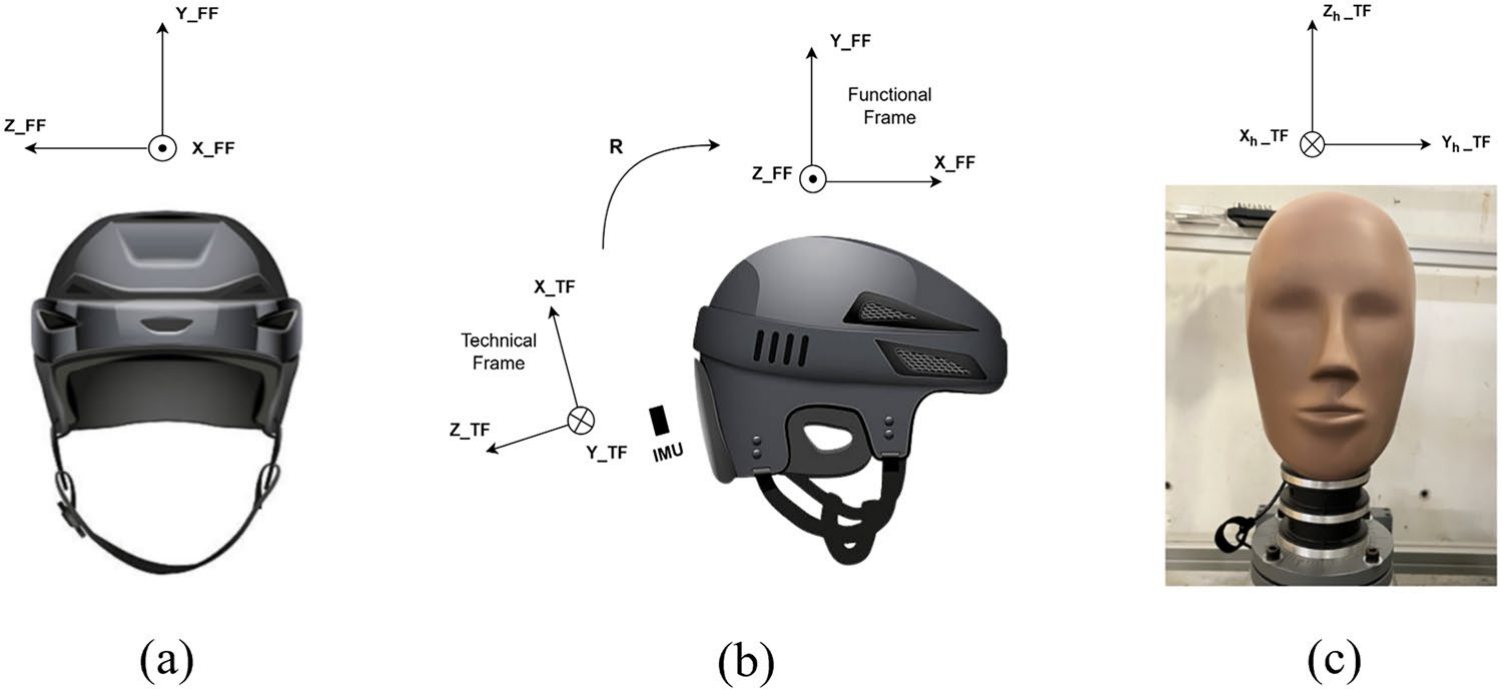
**a** Helmet IMU FF (front view), **b** difference between helmet IMU TF and FF (lateral view), and **c** headform IMU TF

The helmet's Functional Frame (FF) was defined as X_FF = Antero-posterior, Y_FF = Axial and Z_FF = Medial-Lateral (Fig. 2a). A functional calibration was performed by placing the helmet on the headform, and rotating it for 5 s around the Y_FF, followed by 5 s around Z_FF. The gyroscope's raw signal was segmented for the two rotations, and Principal Component Analysis (PCA) was applied on each, to reduce the signal to a single principal component, assumed to be the known axis of rotation. The rotation matrix necessary to transform the helmet IMU measurement from the Technical Frame (TF) into the Functional Frame (FF, Fig. 2b) was then calculated. For the two IMUs to be compared, the headform IMU TF (Fig. 2c) was aligned to the helmet IMU FF. The reference signals (headform) were low-pass filtered at CFC 1000 using the DTS software’s predefined settings.

#### Post-processing

**Fig. 3:**
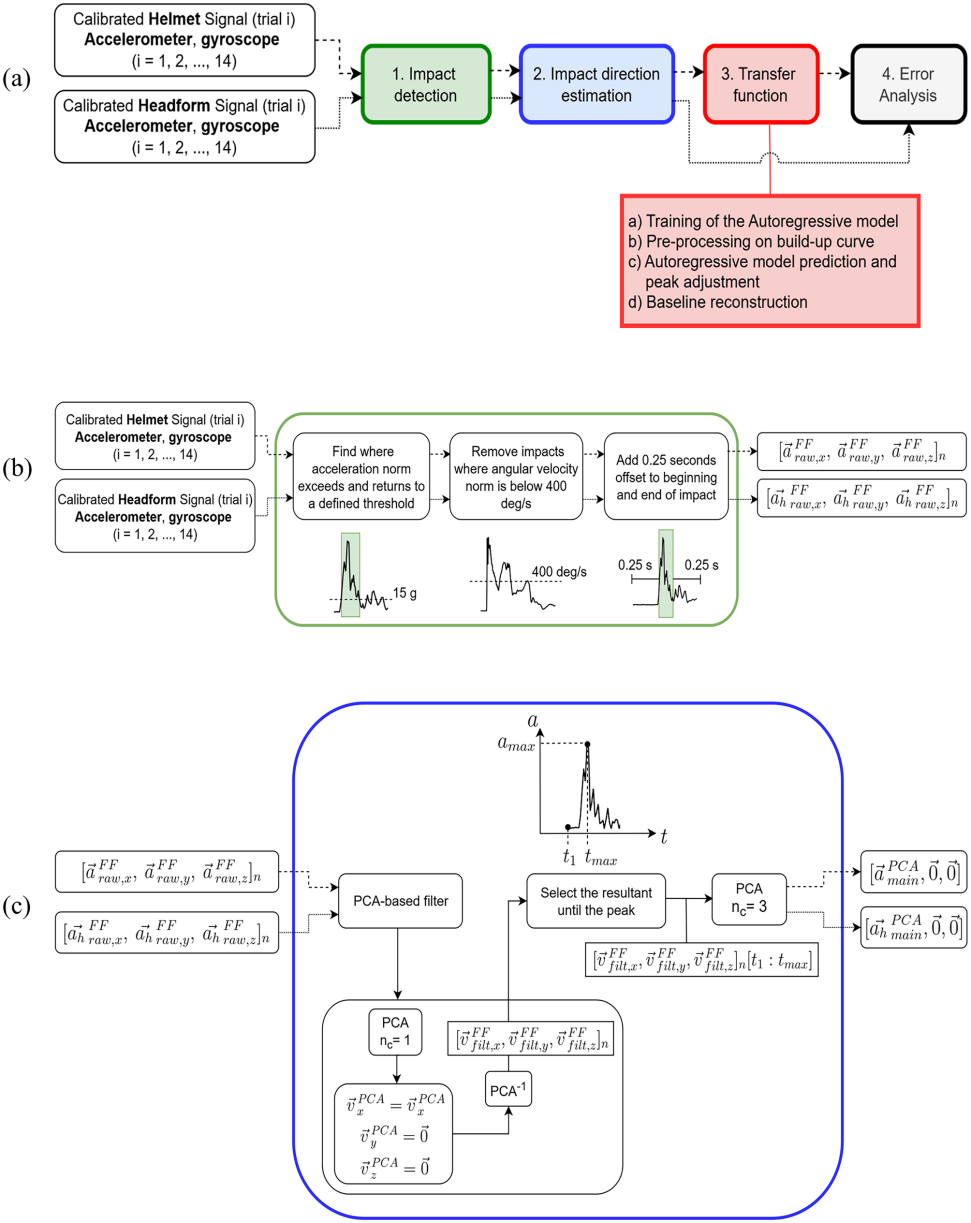
**a** Overview of the processing pipeline, **b** impact detection algorithm, and **c** impact direction estimation algorithm. Given a generic vector ${\overrightarrow{v}}_{ B,c}^{ A}$, A is the frame where the vector is measured, either *FF* Functional Frame or *PCA* Principal Component Analysis (with n_c_ number of components); B indicates whether the signal was filtered (*raw* unfiltered, *filt* filtered) and c is the x, y or z component. For the acceleration measured from the helmet and headform accelerometers ${\overrightarrow{v}}= {\overrightarrow{a}}$ and ${\overrightarrow{v}}= {\overrightarrow{a}}_{h}$, respectively. ${\overrightarrow{a}}_{ main}^{ PCA}$ and ${{\overrightarrow{a}}_{h}}_{ main}^{ PCA}$ are the accelerations projected to the main impact directions for the helmet and headform, respectively. For the steps in the impact direction estimation algorithm (**c**) the vector was kept generic $\overrightarrow{v}$ for clarity in visualization, except for the input and the output, with solid arrows indicating that the steps were applied to both helmet and headform acceleration signals

The post-processing pipeline consisted of three main steps (Fig. 3a), described in the following: (1) Impact detection; (2) Impact direction estimation; (3) Transfer function modeling, applied to the main impact direction. Step 3 was applied only to acceleration data of the helmet IMU.

*Step 1—Impact detection* first, the helmet acceleration data were up-sampled to match the sampling frequency of the headform’s sensor (from 1024 Hz to 10 kHz) and facilitate the comparison of the two signals. The impact detection algorithm (Fig. 3b) worked as follows: (i) the start and end of an impact were calculated as the points where the acceleration norm exceeds and returns to a defined threshold (i.e., 15 g [36]); (ii) a threshold of 400 deg/s was applied to the angular velocity norm to remove false positives impacts caused by events such as sensor removal or helmet adjustments; (iii) an additional ± 0.25 s of the signal was added both pre- and post-impact from the time-points identified at step (i) to capture the entire impact signal waveform.

*Step 2—Impact direction estimation* for each impact, the main direction was estimated using a custom-built algorithm consisting of the following steps (Fig. 3c): (i) data were smoothed using a Principal Component Analysis (PCA)-based denoising method, which worked by first applying PCA with a lower number of components (n_c_ = 1, i.e., first principal component) than the number of input features (equal to 3), and subsequently by projecting the data back to the original frame. The principle is that noise reduction can be achieved by keeping the most significant principal components [37]; (ii) the resultant acceleration was calculated from the filtered data and selected only until the maximum value (*a*_*max*_). The assumption is that at the beginning of an impact (from *t*_1_ to *t*_*max*_, see plot in Fig. 3c), the helmet and headform act similarly, and then the decoupling starts increasing, resulting in noisy signal from the helmet accelerometer. By using only the first part of the signal, the risk of decoupling noise affecting the estimation can be minimized; (iii) the final PCA (n_c_ = 3) was applied, and the estimated impact direction was represented by the first PCA component. Note that, to evaluate the outcome of this impact direction estimation algorithm, a region of impact (θ ± 22.5°) was defined for each direction (see Fig. s2 in Supplementary Material). Since the line of action of the impactor lay on the X_FF–Z_FF plane, only the azimuth angle was considered for the classification. The angle between the estimated direction vectors (x and z components) and the reference vector (see Fig. s2 in Supplementary Material) was calculated, and the impacts were finally classified in one of the pre-defined regions.

The impact detection and direction estimation algorithms were also applied to the headform acceleration, whose outcome (${{\overrightarrow{a}}_{h}}_{main }^{PCA}$ of Fig. 3c) was used as a reference to evaluate the transfer function.

*Step 3—Transfer function modeling* as indicated in Fig. 3a, this processing block includes four stages, labeled Steps 3a–d, which are explained in the following.

**Fig. 4:**
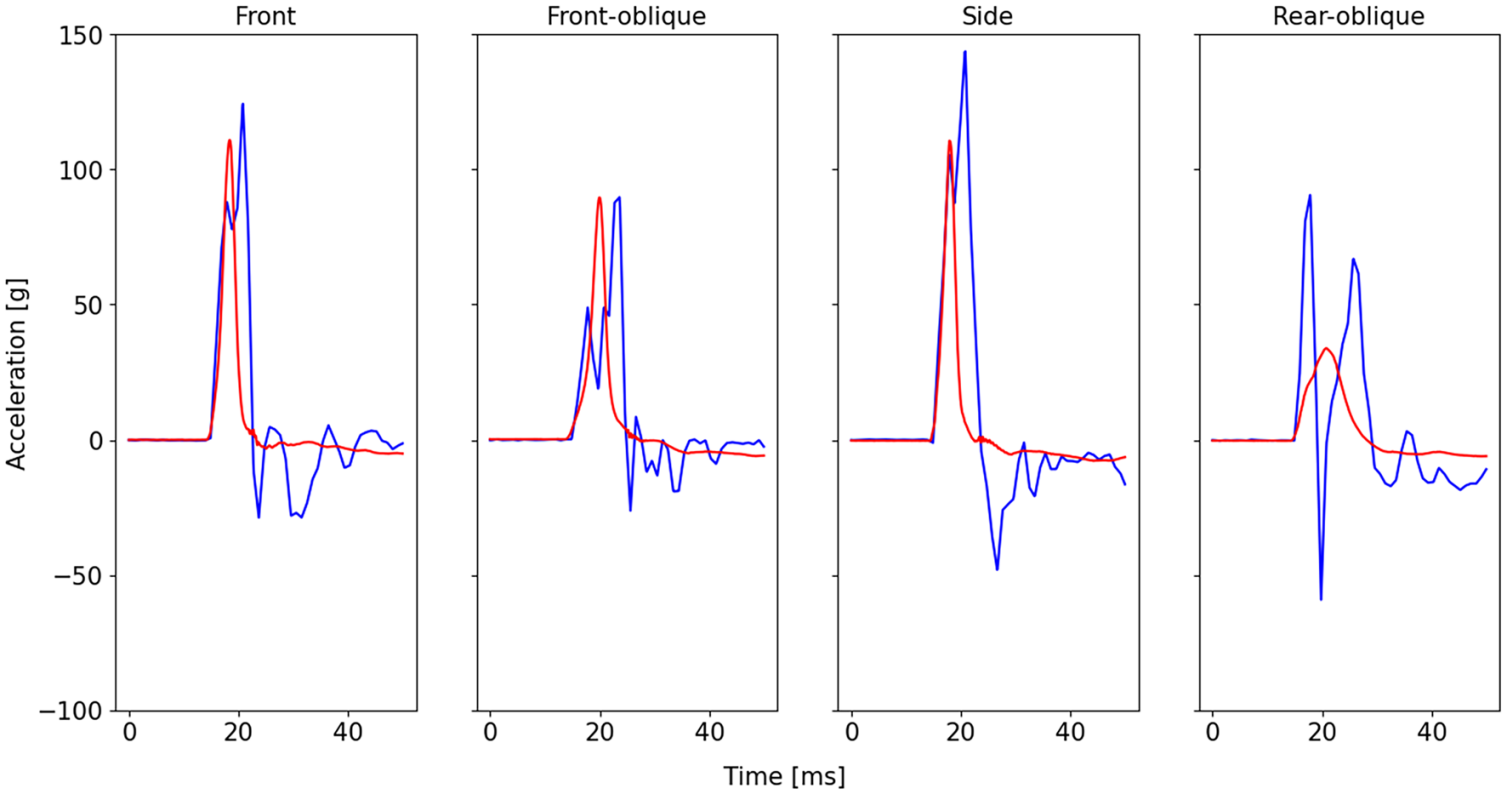
Example of impact signals for front, front–oblique and side (79 J), and rear–oblique (33 J) directions. Helmet and headform IMUs are shown in blue and red respectively. The signals were projected to the main impact direction (PCA frame) and trimmed to 50 ms for a better visualization

*Step 3a—Training of the AutoRegressive models* as previously mentioned, a key point of the transfer function is represented by the implementation of AutoRegressive (AR) models. A model is autoregressive if it predicts future values in a time series by analyzing its historical data and assuming a linear relationship between past and future observations (Eq. 1):

1 $${a}_{t}={c}_{0}+{c}_{1}{a}_{t-1}+ {c}_{2}{a}_{t-2 }+\dots + {c}_{l}{a}_{t-l} + {\varepsilon }_{t},$$

where $a$ is the variable of interest, $t$ is the time instant, ${c}_{i}$ are the model coefficients, ${\varepsilon }_{t}$ is white noise, and $l$ is the lag which defines the number of previous data points to consider. The reasoning behind the use of AR models came from a visual inspection of the linear acceleration of the raw signals, projected to the main impact direction (${{\overrightarrow{a}}}_{main }^{PCA}$ and ${{\overrightarrow{a}}_{h}}_{main }^{PCA}$ of Fig. 3c). Four examples of impact signals in front, side and oblique directions are shown in Fig. 4. Upon closer inspection of the signals, it can be observed that: (i) the headform acceleration is characterized by a Gaussian-like shape; (ii) during the build-up phase of the impact (i.e., the raising part of the curves), the helmet and headform accelerations behave similarly [21], which aligns with the physical intuition that the helmet and head initially move together during the onset of impact*.* A similar behavior has also been observed in previous studies involving mouthguards [38]; (iii) following the build-up phase, the helmet signal shows a “double-peak” behavior that makes the signal descend with a certain delay compared to the headform, especially for front and side directions (see Sect. 3 in Supplementary Material for more details). This specific signal pattern (Fig. 4), which was found to be consistent among the majority of the impacts, could originate from the helmet-to-head decoupling, such as the helmet rebounding or shifting relative to the headform after initial impact. In contrast, the headform, which is rigidly mounted and thus directly coupled to the testing rig, exhibits a single, smoother acceleration peak.

Based on these observations, we selected an autoregressive (AR) modeling approach due to its inherent capability to predict future values in a time series based on past observations. Specifically, our procedure consisted of: (1) training impact-specific AR models using the complete acceleration signals recorded from the headform; (2) applying these AR models to the initial portion (build-up phase) of the helmet acceleration signals, during which helmet–head coupling is maintained. With this method, we aimed to extrapolate and thus reconstruct the portion of the signal affected by helmet–head decoupling and associated distortions.

For a given location, 50% of all impacts for the headform data were randomly selected among both intensities (i.e., 33 J and 79 J, see Table 1) to form the training dataset. This dataset includes also the corresponding signals from the helmet data, which were used for another purpose, as explained in Step 3c. The selected impacts for the headform were randomly reordered and concatenated to create a unique time series that retains the characteristic behavior of the two impact intensities. The approach of concatenating independent time series to generate extended signals, while preserving key dynamic properties, has been previously applied in other fields, such as gait analysis [39].

**Fig. 5:**
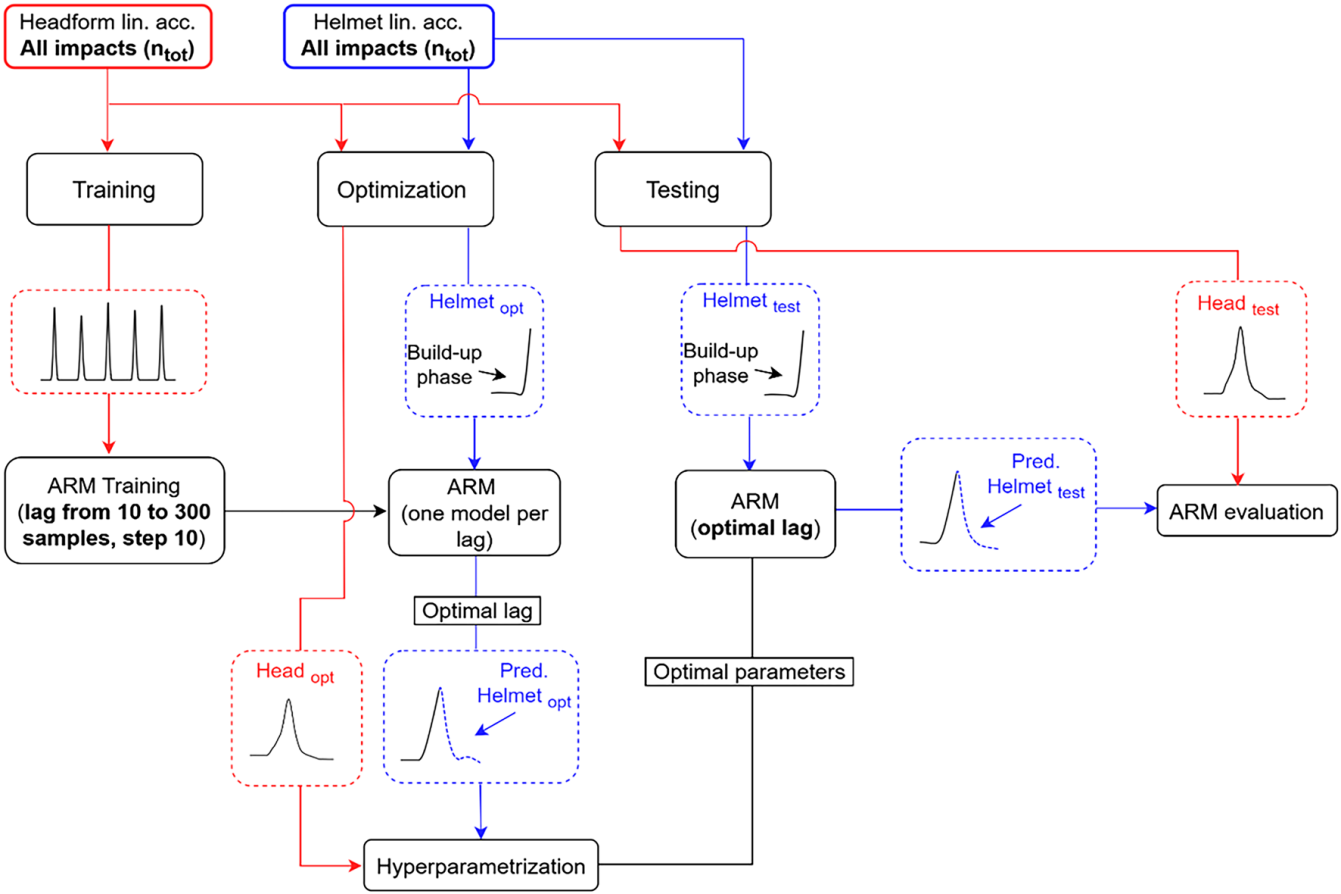
Definition of training, optimization and test datasets for the helmet (blue) and headform (red) data, and their corresponding usage in the AR modeling pipeline. ${\overrightarrow{a}}_{ main}^{ PCA}$ and ${{\overrightarrow{a}}_{h}}_{ main}^{ PCA}$ are the accelerations projected to the main impact directions for the helmet and headform, respectively. *opt* Optimization, *Pred* Prediction, *n*_*tot*_ total number of impacts. Example for a single location

To avoid the model prediction being affected by discontinuities inherent to the concatenation of the impacts, the pre- and post-impact baselines, defined as the segments where the signal is at rest before and after the impact-related peak respectively, were set to zero. The time series was then used to train the AR models and extract the *c*_*i*_ coefficients of Eq. 1. Multiple lags were considered, from 10 to 300 samples, corresponding to a range of 1 to 30 ms, and one model was trained for each lag. The steps to find the optimal lags were applied to a new dataset, named optimization set, which was used also for the hyper-parametrization, as explained in the Step 3c. For each impact location this dataset was created by randomly selecting 25% of the impacts, for the helmet data (not included in the training set). For these impacts the build-up phase was defined as the phase from the start of the impact until the first local maximum, and used as input for the AR models. The optimal lag was then selected by minimizing the Root Mean Squared Error (RMSE), averaged across all the impacts for each location, estimated between the AR predictions and the corresponding headform signals, while maintaining a low computational cost (positively correlated with the lag). Once the final lag was selected for each direction, the final models were used for the evaluation of the transfer function, which was performed on the remaining impacts, defined as the test set [test set = total impacts − (training set + optimization set)] for each location. An overview of the different datasets and their usage in the AR modeling pipeline is illustrated in Fig. 5. In general, the total number of impacts (see Table 1) employed was 140 for training, 70 for validation (optimization), and 87 for testing.

**Fig. 6:**
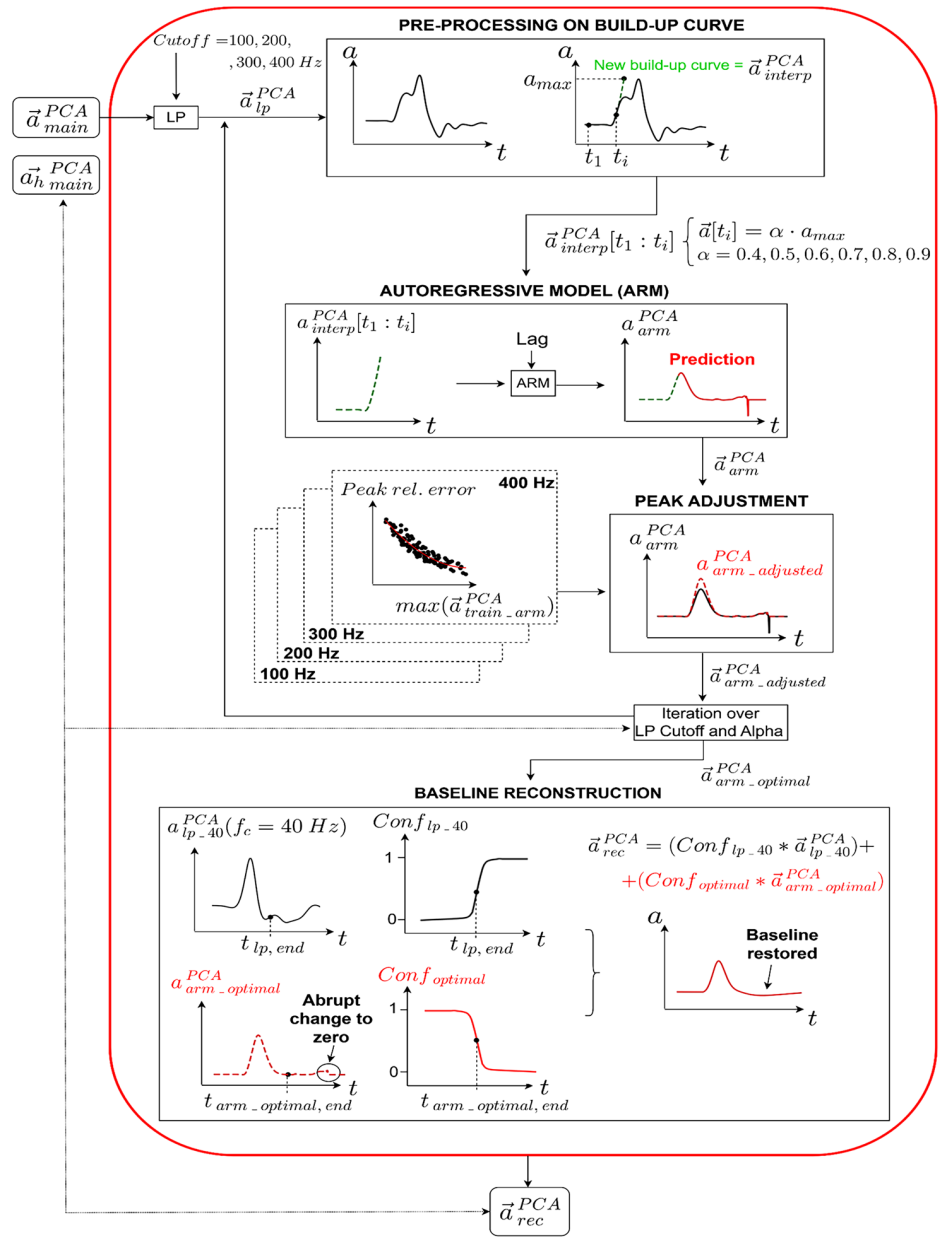
Transfer function modeling pipeline. Given a generic vector ${\overrightarrow{{\varvec{v}}}}_{\boldsymbol{ }{\varvec{B}}}^{\boldsymbol{ }{\varvec{A}}}$; the superscript *A* indicates the frame where the vector is measured (*PCA* Principal Component Analysis); the subscript *B* gives indication on the applied processing (*lp* lowpass filter, *interp* interpolation, *arm* autoregressive modeling, *arm_adjusted* peak adjustment*, arm_optimal* prediction with optimal combination of hyperparameters, *rec* baseline reconstruction). *t*_1_ is the start of the impact trace and *t*_*i*_ is the time corresponding to a specific percentage of the absolute peak (given by α). *t*_*lp_end*_ and *t*_*arm_optimal,end*_ are the times corresponding to the end of the impact for the original signal (filtered at 40 Hz) and the AR prediction, respectively. *LP* Low-Pass, *Conf* Confidence

The implementation of AR models constituted the main block of the transfer function modeling, whose additional processing steps are illustrated in Fig. 6, and described in the following: (i) pre-processing on build-up curve; (ii) autoregressive model and peak adjustment; (iii) baseline reconstruction.

*Step 3b—Pre-processing on build-up curve* the acceleration in the main impact direction was lowpass (LP) filtered with a 4th-order Butterworth filter. Four filter cutoff-frequencies were investigated: 100, 200, 300 and 400 Hz (see Sect. 3 in Supplementary Material for more details). Using the filtered signal, the input to the AR model was obtained as follows: the first local maximum, set to be at least 10% of the absolute peak (in order to discard small irrelevant peaks close to the baseline) was detected and the signal was temporarily selected until this point; a spline interpolation (degree = 2) was applied and extended up to the absolute peak of the raw signal. This interpolation represented the new build-up curve, which was investigated with different peak values, from 40 to 90% (α parameter) of the absolute peak, to investigate how the peak of the input signal affects the AR model prediction.

*Step 3c—AutoRegressive models prediction and peak adjustment* a peak adjustment algorithm was applied to the outcome of the AR models. This algorithm was developed by first building a regression model for each direction and intensity (i.e., using the training set) that related the peak of the AR predictions for the helmet data (referred as ${{\overrightarrow{a}}}_{train\_arm }^{PCA}$) to the peak error (calculated with respect to the corresponding headform data), expressed relative to the AR prediction (Eq. 2).

2 $$Peak\,rel. error= \frac{\left(\text{max}\left({{\overrightarrow{a}}}_{train\_arm }^{PCA}\right)- \text{max}\left({{\overrightarrow{a}}_{h}}_{main }^{PCA}\right)\right)}{\text{max}\left({{\overrightarrow{a}}}_{train\_arm }^{PCA}\right)} *100 .$$

Given a generic new signal (i.e. not included in the training set) ${{\overrightarrow{a}}}_{main }^{PCA}$, the corresponding adjustment ${\Delta }_{peak}$ was calculated (Eq. 3) and used to find the power *x* to which the signal should be raised to compensate for the relative error (Eq. 4):

3 $${\Delta }_{peak}=\frac{\left(Peak\,rel. error\right)}{100}*\text{max}\left({{\overrightarrow{a}}}_{main }^{PCA}\right),$$

4 $${\text{max}\left({{\overrightarrow{a}}}_{main }^{PCA}\right)}^{x}=\text{max}\left({{\overrightarrow{a}}}_{main }^{PCA}\right)+ {\Delta }_{peak}.$$

The reason a ‘power method’ was chosen resides in the possibility of adjusting the whole signal while placing more weight on the samples closer to the peak, resulting in more Gaussian peak shapes. This method has been particularly used in chromatography to reduce the baseline noise and to enhance the resolution of the signals [40].

A grid search was performed on the optimization dataset for each impact configuration (location and intensity) to find the combination of $\text{LP cutoff}$ and $\alpha$ that minimizes the *ErrorScore* (defined as in Eq. 5) between the model prediction (after peak adjustment) and the corresponding headform signal:

5 $$ErrorScore={\text{w}}_{1}\text{* }\frac{\left(\mathit{MeanSlopeError}\right)}{100} +{\text{w}}_{2}* \frac{\left(DurationError\right)}{100}.$$

$ErrorScore$ was set to be the average (${w}_{1}={w}_{2}=0.5$) between the $MeanSlopeError$ and the $DurationError$. The $MeanSlopeError$ is the percentage error (relative to the headform) in the mean slope, which was calculated only for the raising part of the signals, defined from the index where the signal exceeds 5% of the peak (starts raising) to the peak itself. For this segment, the average of the first derivative was calculated. The $DurationError$ is the percentage error (relative to the headform) in the pulse duration, which was defined as the time it takes for the signal to exceed and return to 5% of the absolute peak. The peak error was not included because it would be minimized by the adjustment algorithm. The best combinations of $\text{LP cutoff}$ and $\alpha ,$ derived from the hyperparametrization on the optimization set, were applied to the helmet signals from the test dataset for each impact configuration, and the corresponding prediction was used as input for the final step of the transfer function, the baseline reconstruction (Step 3d).

*Step 3d—Baseline reconstruction* as a reminder, AR models were trained on a zeroed baseline which does not reflect the true acceleration occurring after the peak. Thus, the original post-impact baseline needed to be restored. The reconstruction algorithm worked as follows: the end of an impact pulse (${t}_{end}$ in Fig. 6) was defined as the index where the signal returns to 5% of the absolute peak; the helmet transformed signal (after the peak adjustment) until ${t}_{end}$ is supposed to be representative of the headform’s behavior. On the other hand, as previously mentioned, from ${t}_{end}$ the baseline does not reflect the actual signal. This trend was modeled as a confidence (sigmoid) function equal to 1 until ${t}_{end}$, then decreasing to zero. This function can be seen as a way to give a different weight to each part of the signal, depending on whether that part needs to be preserved or discarded (see Fig. 6, plot *Conf*_*optimal*_). Intuitively, the opposite happens for the unprocessed signal. However, while the baseline of the unprocessed signal follows a similar trend as the headform data, it still can exhibit large spikes, which we addressed by applying a 40 Hz low-pass filter specifically for this purpose (this cutoff was found based on a careful inspection of the signals). A smooth transition from the transformed signal to the post-impact baseline of the original signal (40 Hz lowpass filtered), was obtained by multiplying each signal by its confidence function, and summing the two products.

*Step 4—Error analysis* as mentioned in the Step 3c, the baseline reconstruction was applied to the AR predictions (after peak adjustment) on the test dataset (using directly the optimal combination of $\text{LP cutoff}$ and $\alpha )$.

The output of the baseline reconstruction algorithm was compared with the corresponding headform signals using two features: the Mean Absolute Percentage Error (MAPE, Eq. 6) calculated on the peaks and the RMSE (Eq. 7), calculated on the whole traces, which were trimmed to 40 ms to focus primarily on the impact pulse and reduce the contribution of the pre- and post-impact baselines. The RMSE was averaged across all the impacts for each configuration.

6 $$MAPE=100\frac{1}{N}{\sum }_{i=1}^{N}\left|\frac{\text{max}\left({{\overrightarrow{a}}_{h}}_{main }^{PCA}\right)- \text{max}\left({{\overrightarrow{a}}}_{rec }^{PCA}\right)}{\text{max}\left({{\overrightarrow{a}}_{h}}_{main }^{PCA}\right)}\right| , where\, N=Number\,of\,impacts,$$

7 $$RMSE=\sqrt{\frac{{\sum }_{j=1}^{L}{{{(\overrightarrow{a}}}_{rec }^{PCA}\left[j\right]- {{\overrightarrow{a}}_{h}}_{main }^{PCA}\left[j\right])}^{2}}{\text{L}}} , where\, L=length\left({{\overrightarrow{a}}}_{rec }^{PCA}\right).$$

The same errors were calculated also for the raw helmet linear acceleration (before applying the transfer function) and the lowpass filtered helmet linear acceleration, using the optimal LP cutoff found from the grid search.

#### Evaluation of the Transfer Function on a Different Helmet

To assess the generalization capabilities of the presented methodology, the transfer function was evaluated on a different ice-hockey helmet, Bauer RE-AKT 150. The same IMU used for the CCM helmet was placed in a similar position (back), and a new functional calibration was performed. For this helmet, 96 impacts were conducted in total (24 per direction, with 4 trials per intensity, each consisting of 3 impacts) by following the same procedure. For the back–oblique direction, only the lower intensity was investigated as for the CCM helmet. The entire processing pipeline (impact detection, direction estimation and transfer function) was applied, and each helmet acceleration signal was compared with the corresponding reference from the headform using MAPE and RMSE.

## Results

### Post-processing

#### Impact Detection and Direction Estimation

Figure s3a, b in Supplementary Material show the results of the impact direction estimation algorithm for the helmet and headform sensors. First, it should be noticed that the total number of detected impacts for each direction matches the actual number of impacts conducted (see Table 1). For the headform sensor, all directions were correctly classified. The results for the helmet sensor indicate that almost all directions were correctly classified for the front, front–oblique, and side impacts. In fact, only four right front–oblique impacts were wrongly classified as front. Regarding the back–oblique impacts, 87.5% (21/24) of the directions were correctly classified on the left side, while 23.81% (5/21) for the right side.

#### Transfer Function Evaluation

Regarding the implementation of the transfer function, the first step was the selection of the optimal lag for each impact direction. The results can be seen in Fig. s6 in Supplementary Material. A similar trend was found in all directions, with an initial region of error variability for smaller lags, up to approximately 9 ms, after which the error starts stabilizing, finally reaching a plateau. The optimal lag for the AR models in the three directions was selected to 17 ms, since it is distant from the regions of error variability yet not too high to increase the computational cost of the models.

Once the optimal lags were defined, the final AR models were evaluated on the optimization dataset. Table 2 illustrates the results of the grid search, which was conducted to find the optimal combination of $\text{LP cutoff}$ and $\alpha$ parameters that minimizes the error score (Eq. 5) between the AR model prediction and the reference signal from the headform for each impact configuration (location and intensity).

**Table 2:** Optimal $\text{LP cutoff}$ (Hz) and $\alpha$ parameters that minimize the error score between the AR model prediction and the reference signal, for each impact configuration

Location [intensity (J)]	Error Score	Opt. LP cutoff (Hz)	Opt. α
Front (33)	0.08	200	0.7
Front (79)	0.12	400	0.9
Front–oblique (33)	0.14	100	0.9
Front–oblique (79)	0.19	200	0.9
Side (33)	0.09	200	0.9
Side (79)	0.11	200	0.4
Rear–oblique (33)	0.21	100	0.4

The error score was averaged among all the impacts

*Opt.* Optimal, *LP* LowPass

**Fig. 7:**
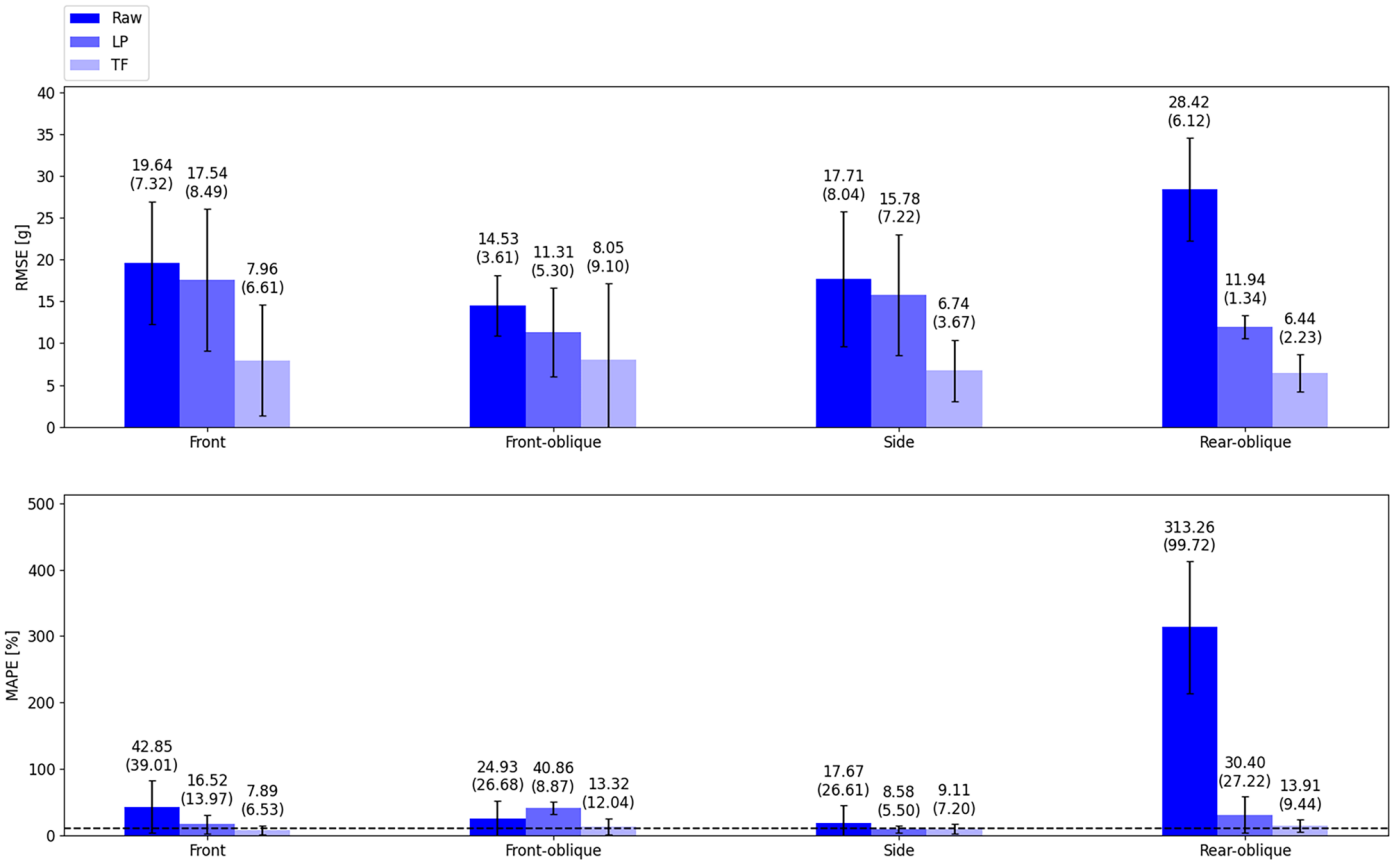
Comparison between unprocessed (Raw), Transformed (TF) and lowpass filtered (LP) helmet signals, for each impact configuration. Mean (± InterQuartile Range IQR) across the impacts for each direction are shown. For the MAPE, the value corresponding to 10% is shown as a black dashed line

For each final combination, the corresponding MAPE and RMSE, calculated on the test dataset, are shown in Fig. 7. For each impact location, the errors were averaged across all the impacts. The results for the unprocessed and the LP filtered (using the optimal cutoff) are also shown.

Looking at Fig. 7, for the RMSE there is a consistent trend, with the transfer function showing the lowest mean error for all the impact directions, followed by the LP filtered and raw signals. Applying the transfer function, compared to the LP filtered, leads to a reduction in the mean error that ranges from 3.26 g (29%) to 9.04 g (57%), with the two bounds corresponding to the front–oblique and side directions, respectively. When comparing the transfer function directly with the raw signal, the highest reduction in the mean error, 21.98 g (77%), is achieved for the rear–oblique direction. The highest variability, 9.10 g, for the transformed signal was found on the front–oblique direction, compared to both unprocessed and LP filtered signals (3.61 g and 5.30 g, respectively). Regarding the MAPE, the transfer function exhibits the lowest error for the front (7.89%), front–oblique (13.32%) and rear–oblique (13.91%) directions.

The corresponding reductions in the error are approximately 35%, 12% and 299% for the raw signal, while for the LP filtered 9%, 27.54% and 16.49%. Instead, for the side impacts, the LP filtered shows a higher performance, leading to a drop in the error compared to the transfer function of 0.53%. In all the directions the transformed signal showed lower variability compared to the unprocessed signal, with the largest difference found for rear–oblique impacts (90.28%). Compared to the LP filtered signals, the transfer function reduced the variability by 7.44% for front impacts and 17.78% for rear–oblique impacts. However, for front–oblique and side impacts, the LP filtered signals exhibited lower variability, with a reduction of 3.17% and 1.7%, respectively.

#### Evaluation of the Transfer Function on a Different Helmet Model

The results of the transfer function for the CCM Tacks 910 and Bauer RE-AKT 150 helmets are summarized in Table 3. It can be seen that for the Bauer helmet, the MAPE error (averaged across the impacts for each direction) is remarkably higher compared to the CCM helmet, ranging from approximately 16.90% (rear–oblique direction) to 78% (side direction). Regarding the RMSE, applying the transfer function to the Bauer helmet leads to an increase in the mean error by 5.39 g (67%), 8.84 g (109%) and 8.74 g (129%) for the front, front–oblique and side directions, respectively, and a decrease of 1.88 g (29%) for the rear–oblique direction. The results of the impact detection and direction estimation algorithms for the Bauer helmet can be seen in Supplementary Material (Fig. s3c, d).

**Table 3:** MAPE (%) and RMSE (g) between the transformed helmet linear acceleration and the corresponding reference from the headform, for the two ice hockey helmets (CCM Tacks 910 and Bauer RE-AKT 150)

Location	CCM MAPE	Bauer MAPE	CCM RMSE	Bauer RMSE
Front	7.89 (6.53)	67.68 (27.50)	7.96 (6.61)	13.35 (12.93)
Front–oblique	13.32 (12.04)	83.93 (26.72)	8.05 (9.10)	16.89 (9.41)
Side	9.11 (7.20)	78.68 (14.81)	6.74 (3.67)	15.48 (9.08)
Rear–oblique	13.91 (9.44)	16.90 (7.58)	6.44 (2.23)	4.56 (1.14)

For each direction, the errors represent the mean (± InterQuartile Range IQR) across all the impacts

## Discussion

The impact direction estimation algorithm showed the lowest performance for the back–oblique direction. Analysis of the impacts in the frequency domain revealed that the back–oblique direction was characterized by the highest mean frequency of helmet acceleration (see Supplementary Material, Sect. 3). This is understandable, as this location is in close proximity to the helmet’s IMU making the sensor more sensitive to the shock. This increased sensitivity can result in noisy behavior, potentially affecting the accuracy of the estimation.

For the other directions, the results of the grid search (Table 2) indicate that the optimal $\text{LP cutoff}$–$\alpha$ combination depends not only on the impact location but also on the severity (intensity). For the LP cutoff, this dependence can be explained by the different attenuation due to the nonlinear response of the helmet’s protective materials. This outcome suggests that a single AR model could be developed for each direction, and the prediction can be subsequently adapted to the different intensities by adjusting the $\text{LP cutoff}$ and $\alpha$ parameters. Additionally, given the higher mean frequency observed for oblique impacts along the main axes (see Sect. 3 in Supplementary Material), we anticipated that these directions would be more affected by the filtering process. Interestingly, Table 2 shows that for the oblique impacts (front and rear, 33 J), the error score is minimized using the lowest cutoff frequency, 100 Hz.

The results from the final AR models evaluation (Fig. 7) indicate that the developed transfer function consistently reduces the error between the helmet and the headform signals compared the LP-filtered helmet signals, suggesting that usual processing methods might not be sufficient to achieve the same level of accuracy. An exception is observed for the MAPE estimated for the side direction, where the LP filter shows a 0.5% reduction in error relative to the transfer function. However, in terms of RMSE estimated for the same direction, the transfer function leads to 9.04 g (57%) reduction over the LP filtered, suggesting that both errors should be considered when evaluating the overall impacts, a factor that has been largely overlooked in previous studies. For both MAPE and RMSE the highest variability was found for the front–oblique impacts, which may be attributed to the varying degree of decoupling between the two impact intensities.

By comparing our results with those of Allison et al. [18, 20], which specifically focus on ice hockey, we can draw the following conclusions: the lowest error measured with the gForce Tracker (GFT) sensor systems was 96.8% [20], observed when the IMU was placed on the inside top of the helmet. While this error was calculated by averaging across all impact directions, which differs from our approach, it is roughly seven times higher than the largest error observed for our transfer function, which was 13.91%, found for the back–oblique impacts (Table 3). Regarding the HIT system [18], the average absolute error that we obtained using our transfer function is 14% and 17% lower in the side and rear–oblique directions, respectively, which are the directions common to both studies. Additionally, for the same locations, we achieved lower variability, with reductions of 0.8% for the side and 12.56% for the rear–oblique impacts. The HIT system has also been investigated in the context of American football by Rowson et al. [21] and Jadischke et al. [22], although their studies examined different impact locations and velocities. In Rowson et al. study, the relative peak error for linear acceleration was found to be 1% ± 18% when combining all the directions. As this is a relative error, it is not directly comparable to the absolute percentage errors we reported. Regarding the RMSE, the use of a different temporal sequence length for the temporal sequence in our study makes the comparison difficult. In Jadischke’s study [22], an absolute percentage peak error greater than 15% occurred in 55% of all the impacts for linear acceleration. In contrast, using our transfer function, the peak error never exceeds 15% on average across all the impact directions.

In our study we also assessed the generalization capabilities of our transfer function, by applying it to a different ice-hockey helmet. The results presented in Table 3 show a significant decrease in the performance of the AR models in this case. This decline can be attributed to variations in impact dynamics between helmet models (see Fig. s7 in Supplementary Material), influenced by factors such as helmet shape, inner foam configuration, and material properties. Since the AR models were developed using headform signals recorded with the CCM helmet, these findings indicate that the models must be tailored not only to the impact direction but also to the specific helmet type. More advanced modeling approaches, such as machine learning or deep learning techniques, could potentially improve the robustness and generalizability of predictions across different helmet designs.

However, our results emphasize the importance of analyzing helmet–headform decoupling, which can provide valuable insights for a more accurate assessment of head impact severity, as well as for a better optimization of helmet design. In fact, quantifying the discrepancies between helmet and headform motion during impact allows for the identification of conditions under which excessive relative motion occurs. Such information can inform the optimization of helmet components—including the fit system, padding, and shell–liner interface—to reduce relative motion and ensure better coupling between the helmet and the head, thus improving the overall safety and performance.

### Limitations

This study has several limitations that should be considered. The first challenges stem from the in-lab data collection. While the impact tests were designed to closely replicate head impacts during ice hockey games, a study conducted by Liu et al. [41] demonstrated that using 50th percentile adult male headforms may not be sufficient to generalize across a diverse population. For instance, the Hybrid III used in this study presents simplified facial features and does not include mandible and nape [42]. Moreover, it is covered with a vinyl plastisol skin [43], which alters the coefficient of friction between the helmet and the headform [44]. Future research could explore the use of additional layers, such as skull caps or wigs, to represent different levels of fit, as well as investigate the effects of using multiple helmet sizes.

The impact velocities used in this study represent another limitation to the generalizability of the results. The methodology was based solely on two impact intensities, with rear–oblique impacts tested at only one intensity, highlighting the need to develop new models that can accommodate a broader range of severities. One potential approach could be to define the AR models, as well as the $\text{LP cutoff}$–$\alpha$ parameters, for discrete reference intensities (as shown in this study), followed by the implementation of predictive models capable of interpolating or extrapolating these parameters for intermediate or untested impact levels. Additionally, the magnitudes of the impacts recorded were obtained using a specific impact mass and neck's stiffness. These two features can generally be adjusted, and we encourage future studies to explore their effect on the impact dynamics. Furthermore, this study utilizes a biased neckform system, which is characterized by a more constrained response due to its asymmetrical compliance [45]. Investigating the use of unbiased neckforms could provide a more representative assessment of dynamic responses during impacts.

Regarding the data processing, the first limitation lies in the impact direction estimation algorithm. As previously explained, the classification algorithm relied on calculating the azimuth angle on the X_FF–Z_FF plane. However, for a more comprehensive assessment of the impact location, the elevation angle should also be included in the analysis. Additionally, the boundaries defining the impact regions (Fig. s2 in Supplementary Material) are inherently subjective (for instance, it could be argued that the front region should be narrower). Caution is necessary when interpreting the results, particularly regarding potential discrepancies between the helmet and headform directions within the same region. Another limitation in the processing is related to the model’s adaptability. In particular, additional features beyond those currently considered, or alternative AR lag values, could be explored to better capture the complexity of the signals and improve model tuning. Likewise, alternative filtering methods—beyond the low-pass filters currently applied, including the specific filter used for baseline reconstruction, might be explored.

Finally, the transfer function presented in this study was specifically developed for data recorded from the accelerometers. However, analyzing acceleration alone is insufficient, as rotational motions are also considered significant contributors to brain injuries. Therefore, investigating the application of similar techniques to the angular velocity signals could prove valuable.

## Supplementary Information

Below is the link to the electronic supplementary material.

Supplementary file1 (PDF 3049 kb)

## Data Availability

The data are available from the corresponding author upon reasonable request.
