# Supplementary material for: Helmet–Head Decoupling in Ice Hockey Impacts: An In-lab Exploratory Study Using Autoregressive Modeling of Acceleration Data Measured from a Helmet-Mounted Inertial Measurement Unit (IMU)
Source: Ann Biomed Eng. 2025 Sep 25;53(11):3141–55. doi: 10.1007/s10439-025-03848-2 (PMC12575489; doi:10.1007/s10439-025-03848-2)
Supplement: Supplementary file 1 — Supplementary file1 (PDF 3049 kb) [file 10439_2025_3848_MOESM1_ESM.pdf]

Supplementary Material

1. Pendulum impactor

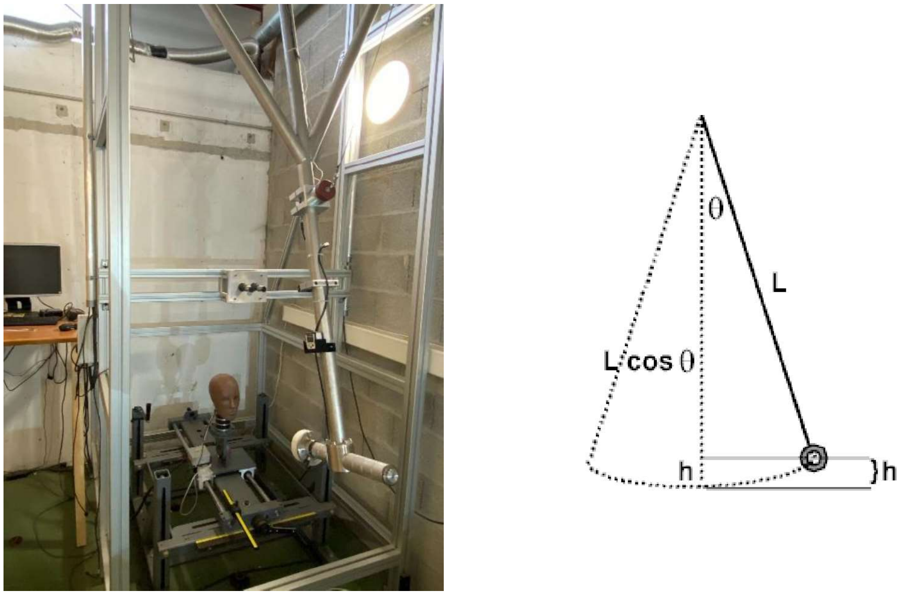

Figure s1: Pendulum impactor (left) with the corresponding simplified schema (right)

The pendulum arm angles, corresponding to the impact velocities, were derived as follows: at the beginning of the impact test, the pendulum mass is raised to a certain height *h* and held at rest. At this point, the system possesses gravitational potential energy. Instead, just before the impact (after the arm is released) we can assume that the system reaches its maximum kinetic energy. From the conservation of energy (applied to the center of mass of the pendulum CoM):

$$v_{CoM} = \sqrt{2 \cdot g \cdot h_{CoM}} = \sqrt{2 \cdot g \cdot L_{CoM}\left(1 - cos(\theta)\right)} \qquad (1)$$

The velocity for the impact mass can be derived as:

$$v_{impactMass} = \dot{\theta} \cdot L_{impactMass} = \frac{v_{CoM}}{L_{CoM}} \cdot L_{impactMass}$$

$$= \sqrt{2 \cdot g \cdot L_{CoM}\left(1 - cos(\theta)\right)} \cdot \frac{L_{impactMass}}{L_{CoM}} \qquad (2)$$

From equation 2 the pendulum arm angle can be calculated as:

$$\theta = cos^{-1}\left(1 - \left(\frac{v_{impactMass}}{L_{impactMass}}\right)^2 \frac{L_{CoM}}{2g}\right) \qquad (3)$$

The associated impact energies can be obtained from the conservation of energy applied to the whole mass (system pendulum + impact mass):

$$E_{impact} = E_{pot} = m_{total} \cdot g \cdot h_{CoM} = m_{total} \cdot g \cdot L_{CoM} \cdot \left(1 - cos(\theta)\right) \quad (4)$$

The equations were implemented with the following values:

$m_{impactMass} = 6.7$ [kg]

$m_{arm} = 13.5$ [kg]

$m_{total} = 20.2$ [kg]

$L_{CoM} = 1110$ [mm]

$L_{arm} = 1848$ [mm] $= L_{impactMass}$

Values corresponding to the dimensions of the pendulum's components were obtained from the corresponding CAD file.

2. Impact detection and direction estimation algorithm

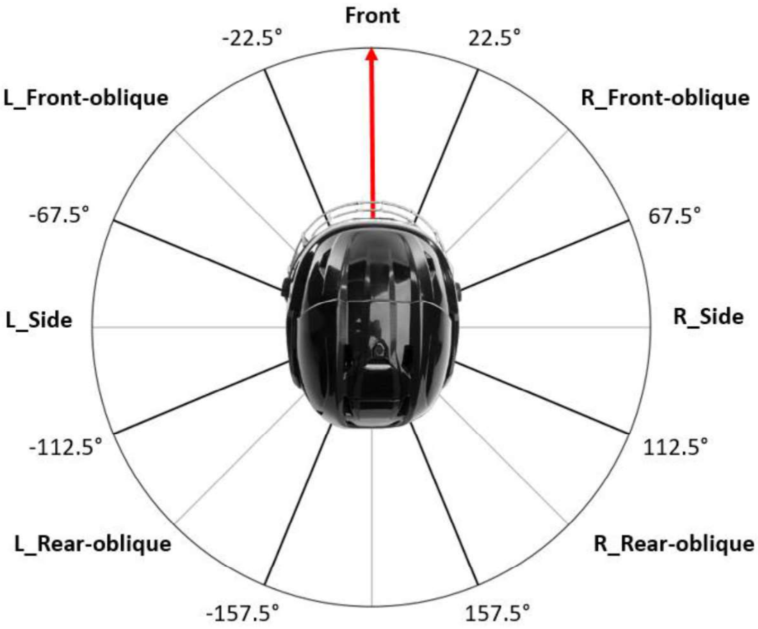

Figure s2: Impact regions defined for the direction estimation algorithm (top view). The reference vector (front direction) is indicated with the red arrow.

	CCM			Bauer		
**Location**	**Precision**	**Recall**	**f1-score**	**Precision**	**Recall**	**f1-score**
Front	0.95	1	0.98	0.77	1	0.87
L_Front-oblique	1	1	1	1	1	1
R_Front-oblique	1	0.9	0.95	1	0.42	0.59
L_Side	0.94	1	0.97	1	1	1
R-Side	0.69	1	0.82	1	1	1
L_Rear-oblique	1	0.88	0.93	1	0.58	0.74
R_Rear-oblique	1	0.24	0.38	1	0.58	0.74

Table s1: Precision, Recall and f1-score for the two helmets CCM Tacks 910 and Bauer RE-AKT 150. Abbreviations: L = Left; R = Right; obl = oblique.

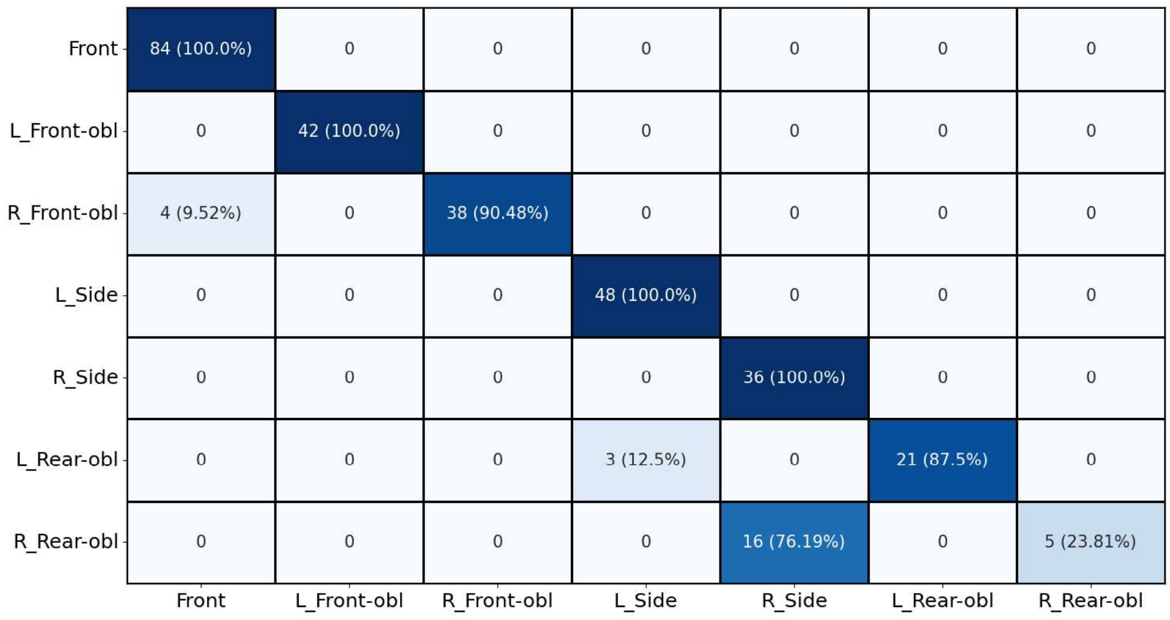

(a) Helmet IMU

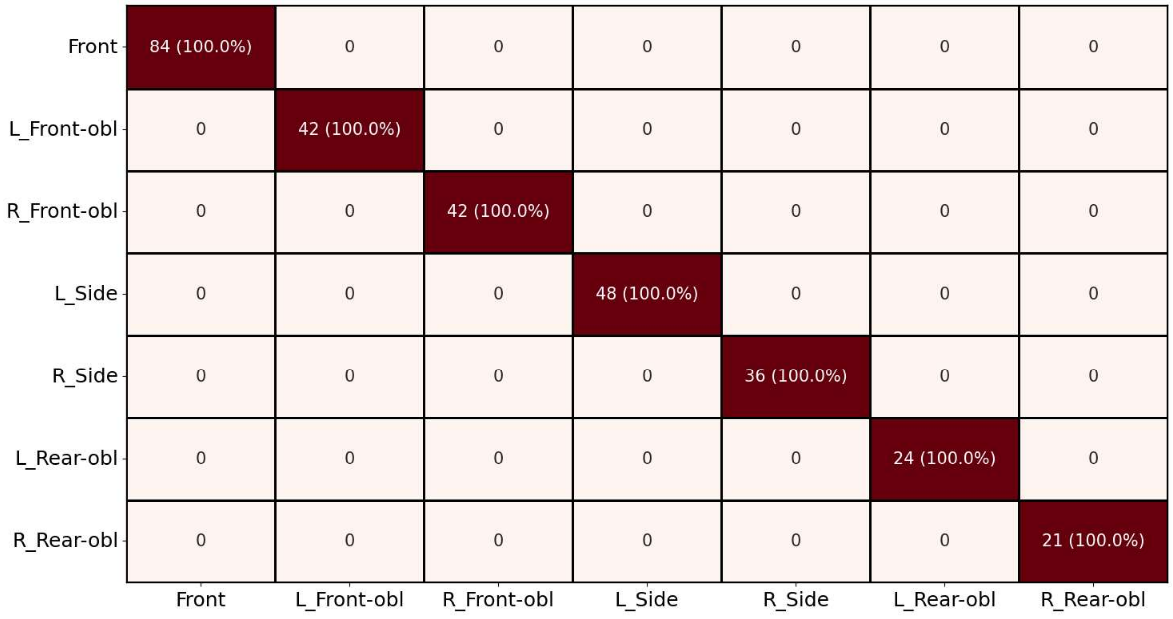

(b) Headform IMU

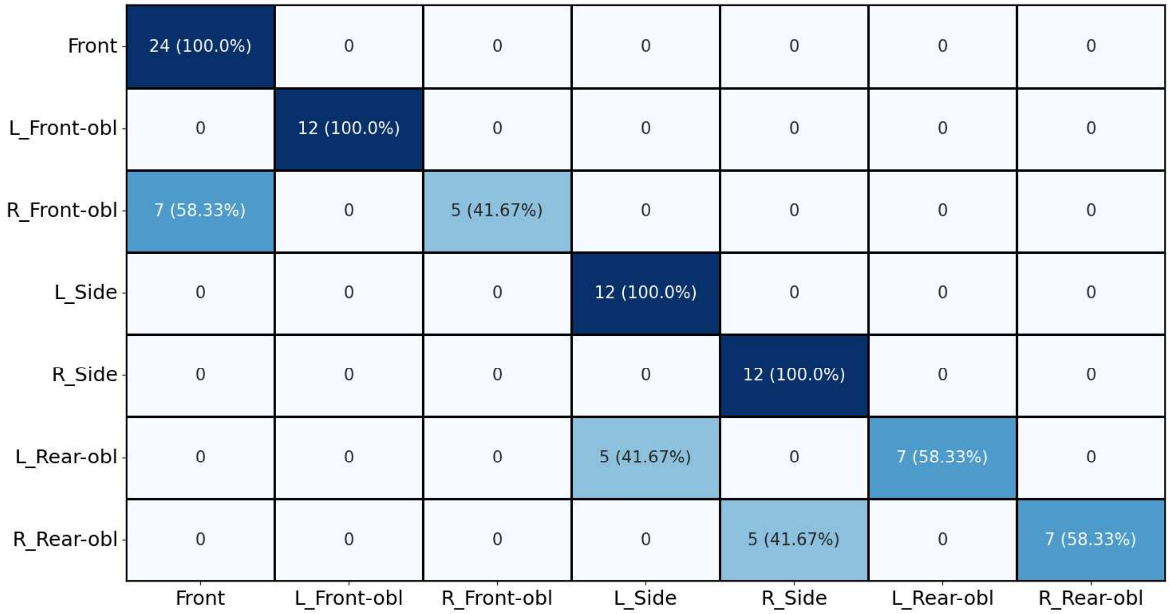

(c) Helmet IMU

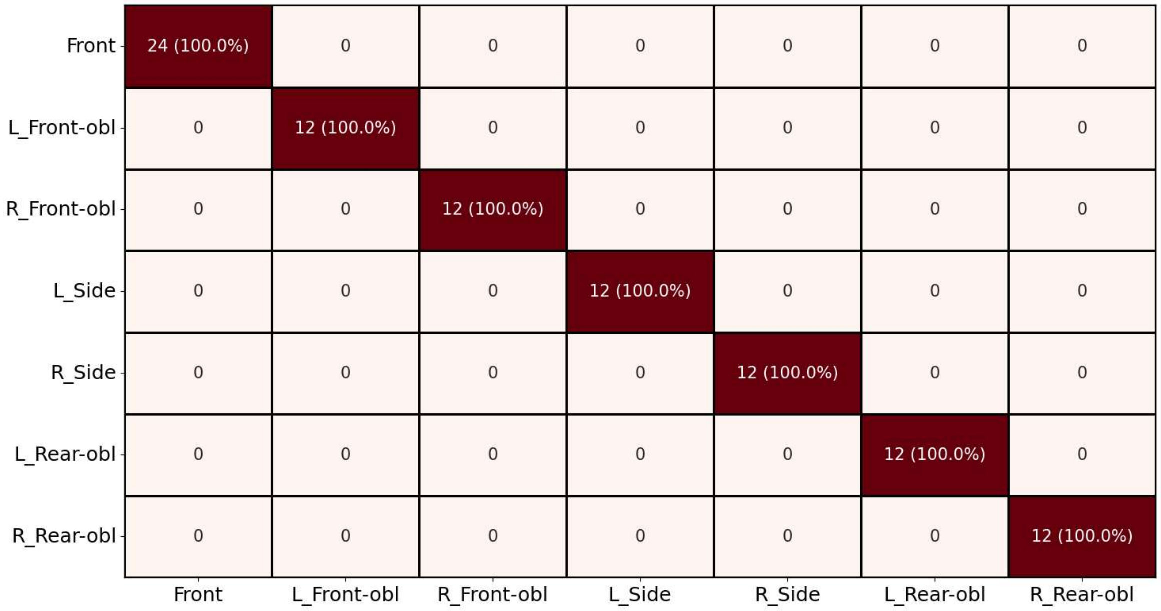

(d) Headform IMU

Figure s3: Confusion matrices showing the results of the impact direction estimation algorithm for the helmet CCM Tacks 910 (s3a and s3b) and Bauer RE-AKT 150 (s3c and s3d). Abbreviations: L = Left; R = Right; obl = oblique.

3. Analysis in the frequency domain

Figure 4 shows that during the build-up phase, the helmet and headform linear acceleration profiles behave similarly. However, for the oblique impact, the helmet (blue curve) shows a steeper raising phase. This behaviour was found to be more emphasized for the 33 J intensity impacts (pendulum arm 35 degrees). It is noteworthy that the greatest decoupling (relative movement between helmet and head) was observed during oblique impacts. We hypothesized that a steeper build-up phase might be caused by high-frequency components in the signal representing the movement of the helmet. This was investigated more in detail by first calculating the power spectrum for helmet and headform signals in the Functional Frame for each impact configuration (Figure s4).

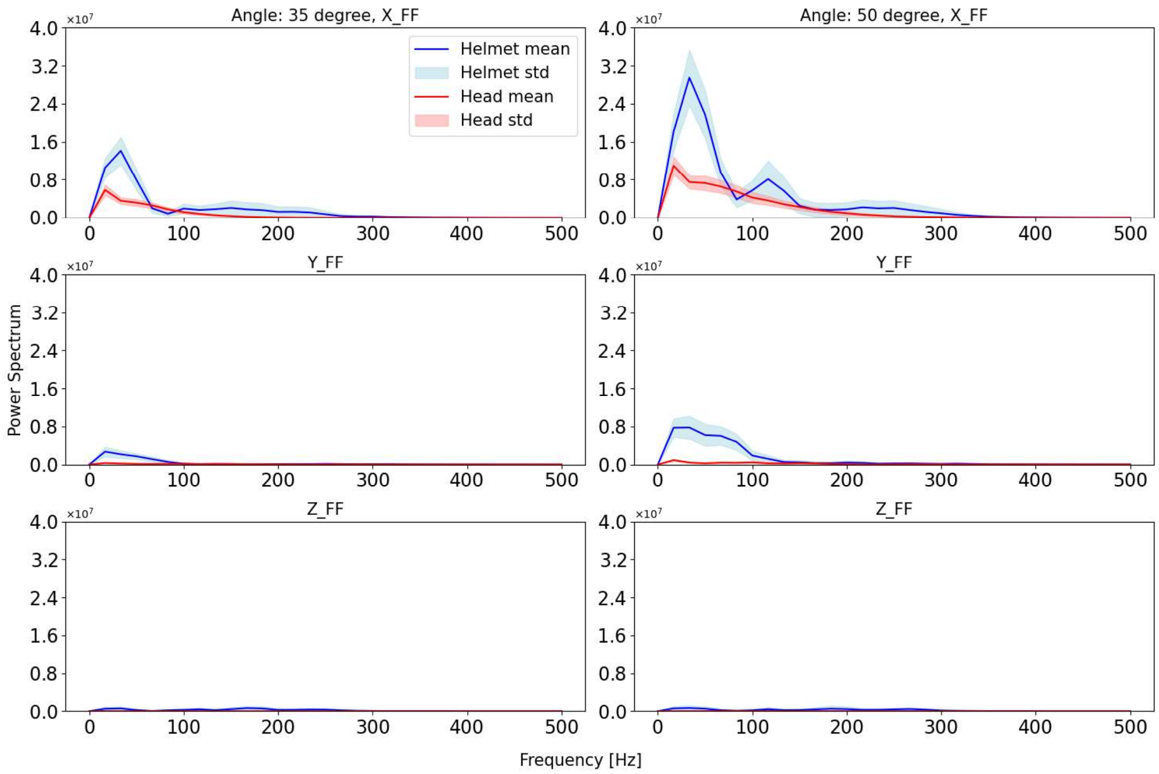

(a) Front

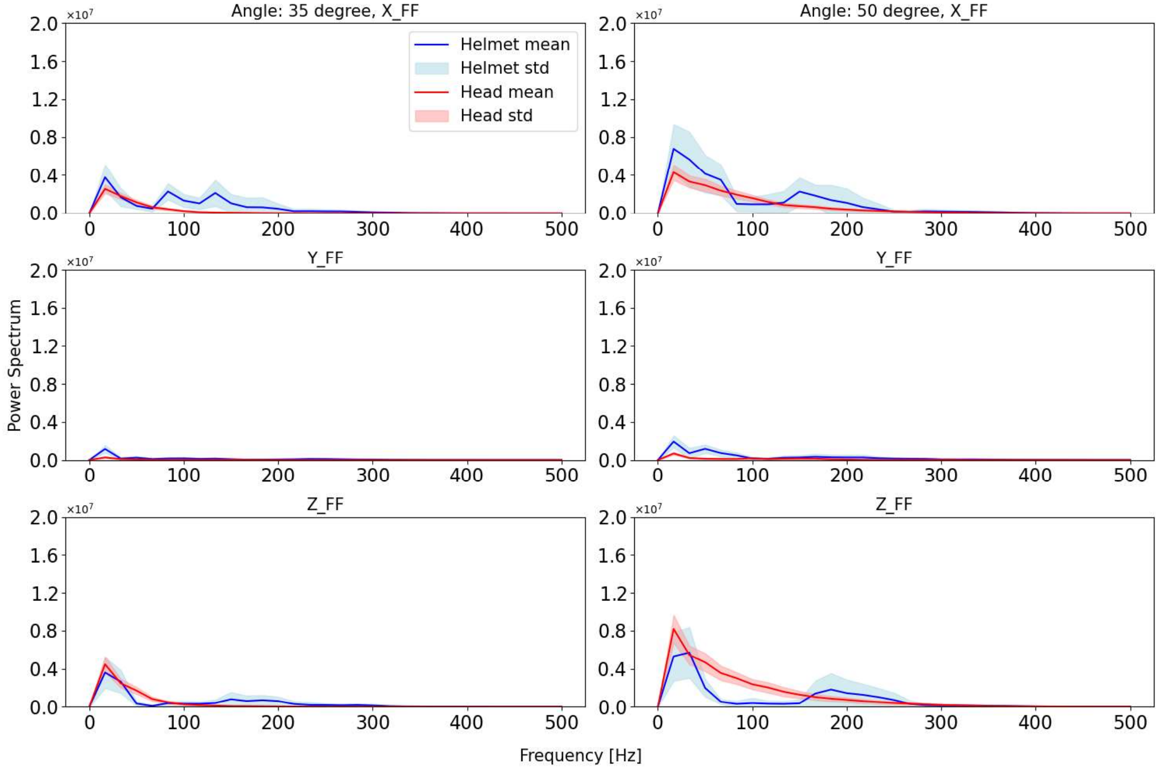

(b) Front-oblique

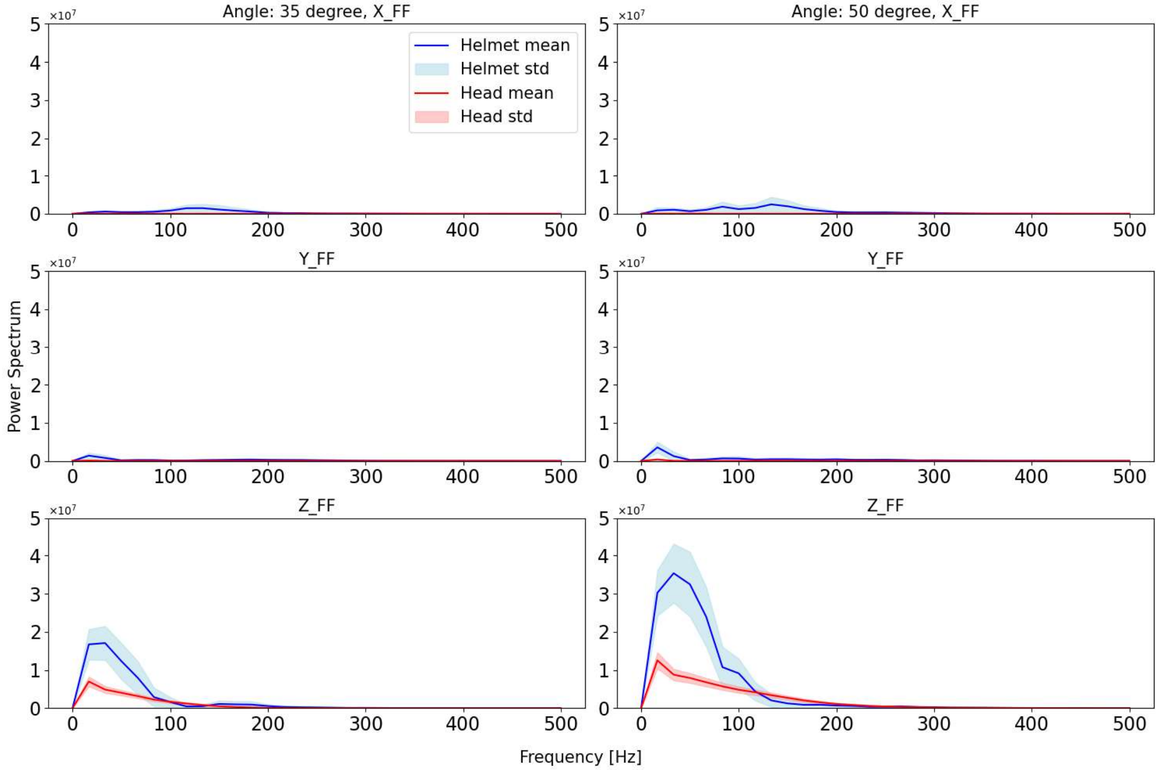

(c) Side

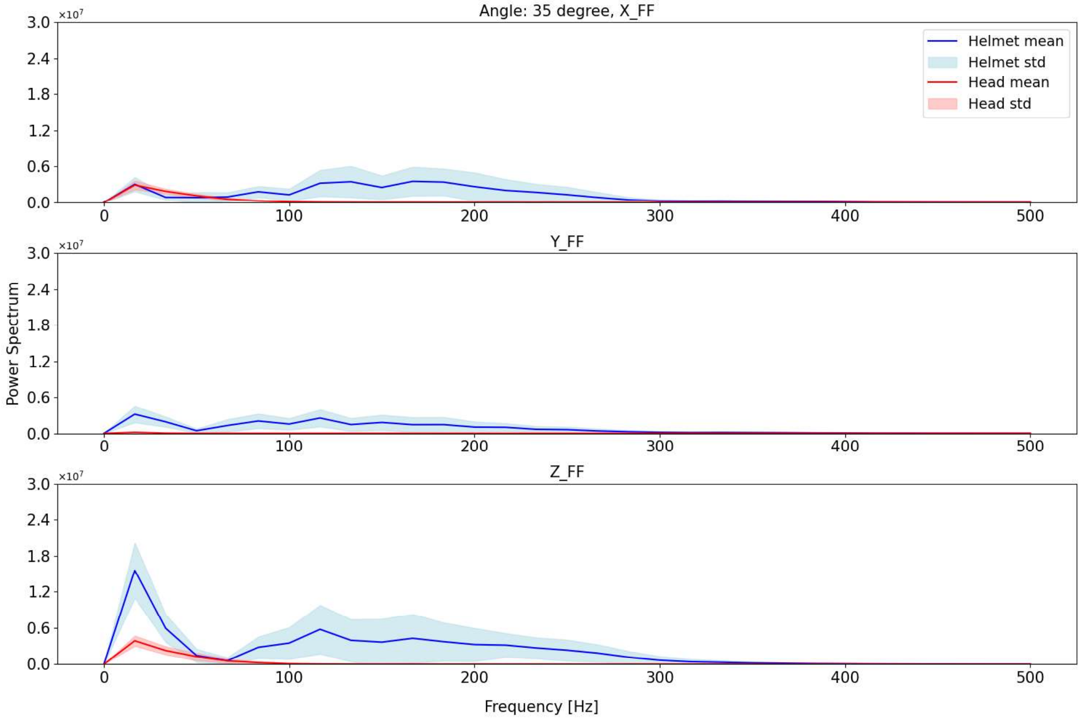

(d) Rear-oblique

Figure s4: Power spectrum (mean ± standard deviation) for each impact configuration.

Recalling the Functional Frame of the helmet IMU, which is the main reference system, the front impacts occur along the X_FF axis, the side impacts along the Z_FF and the oblique impacts on the X_FF–Z_FF plane. From Figure s4, it can be seen that for each impact location, the helmet shows high-frequency components, compared to the headform, in the axes that do not follow the main direction, which can represent noise due to the helmet movement (relative to the head) or vibrations. Interestingly, for the front-oblique impacts (especially pendulum arm 35 degrees), this discrepancy with the headform can also be found in the main axes and could be related to the steeper build-up phase previously described. The back-oblique impacts are even more evident. Indeed, the back-oblique location is near the helmet IMU, making the sensor much more sensitive to the shock. For this reason, the 79 J impacts were not conducted in this case. After having analyzed the power spectrum, the mean frequency, which is one of the most used features in the frequency domain [46], for each impact configuration was calculated using the following formula [46]:

$$\bar{f} = \frac{\sum (P_s * f)}{\sum P_s} \qquad (s1)$$

where P_S is the power spectrum and f the frequency array. Results are illustrated in the Table s2.

Location (Intensity [J])	Head X_FF	Helmet X_FF	Head Y_FF	Helmet Y_FF	Head Z_FF	Helmet Z_FF
Front (33)	56 ± 2	81 ± 18	113 ± 8	73 ± 19	112 ± 38	157 ± 23
Front (79)	79 ± 5	84 ± 15	115 ± 8	71 ± 11	138 ± 48	174 ± 32
Front-oblique (33)	44 ± 2	102 ± 44	75 ± 21	106 ± 20	42 ± 3	99 ± 21
Front-oblique (79)	81 ± 6	94 ± 22	109 ± 13	109 ± 19	83 ± 8	105 ± 22
Side (33)	85 ± 14	135 ± 14	90 ± 36	128 ± 30	58 ± 4	54 ± 7
Side (79)	112 ± 23	139 ± 112	88 ± 28	121 ± 29	83 ± 5	59 ± 6
Rear-oblique (33)	37 ± 3	158 ± 37	57 ± 16	132 ± 15	35 ± 2	123 ± 20
**Mean value**	58	113	92	105	79	110

Table s2: Mean frequency [Hz] (mean ± standard deviation) for each impact. Configuration

From Table s2, looking at the main direction axes, X_FF for the front, Z_FF for the side and X_FF-Z_FF for the oblique impacts, it should be noticed that overall, the absolute difference between the mean frequency of the helmet and headform is higher in the oblique directions. This is in accordance with the fact that for these impacts, the greatest decoupling was observed, and we believe it could also explain the presence of the steeper build-up phase, as previously described. In order to decrease the discrepancy with the headform during the build-up phase, a lowpass 4th order Butterworth filter was added as the first step of the transfer function. In fact, using a lowpass filter, the slope of the signal during the raising part is decreased due to the smoothing (an example is shown in the Figure s5). Four different cutoffs were investigated, from 100 (which is close to the mean frequency averaged among all the impact configurations) to 400 Hz (step 100). Although the filter was introduced mainly for the oblique impacts, it was applied also to the other directions in order to have a more standardized pipeline, although we expect the oblique impacts to be more affected.

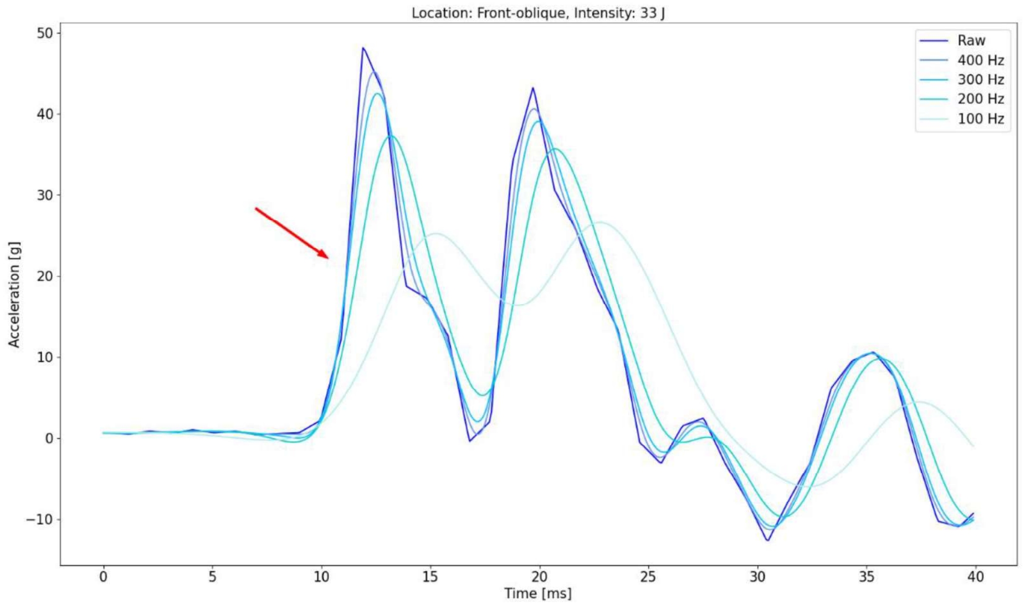

Figure s5: Effect of lowpass filter on the slope of the build-up phase (red arrow). The signal is projected to the main impact direction.

4. Results of the AR models validation: optimal lag selection

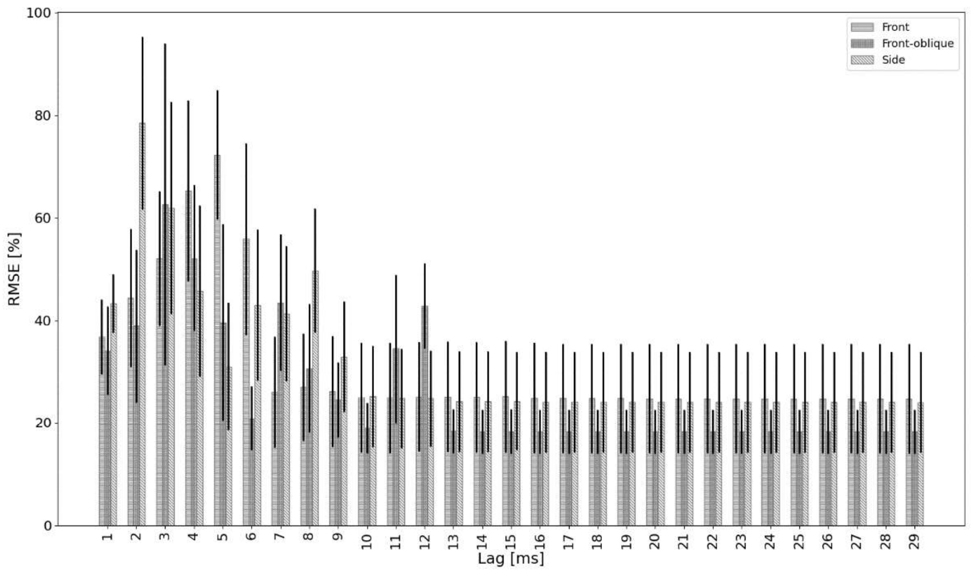

Figure s6: Results of the AR models validation, used to select the optimal lag in each impact direction.

5. The effect of the helmet type on the impact dynamics

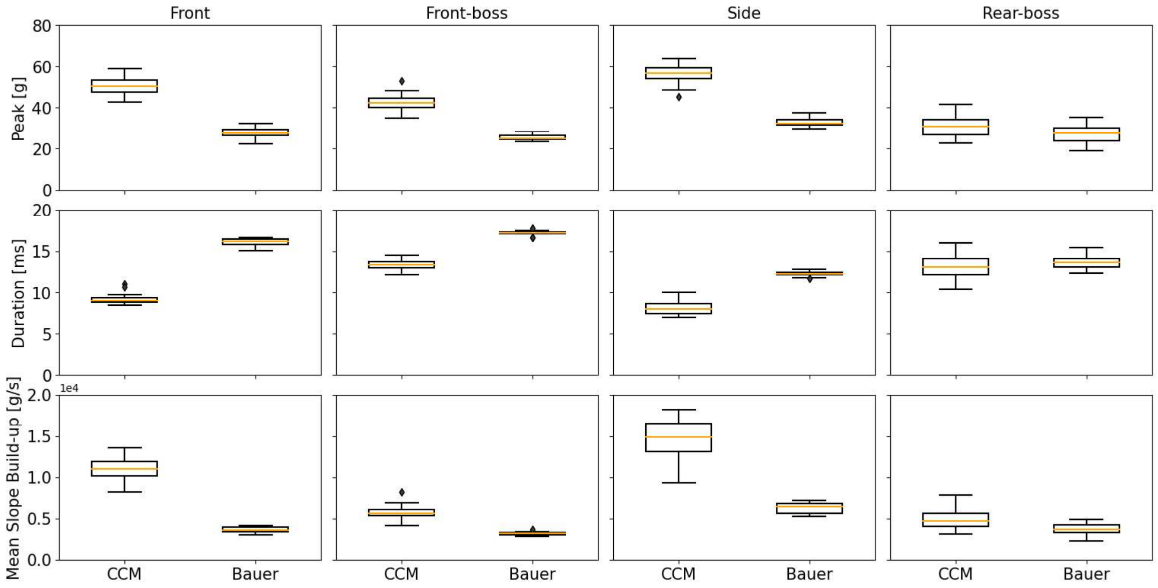

(a) 33 J

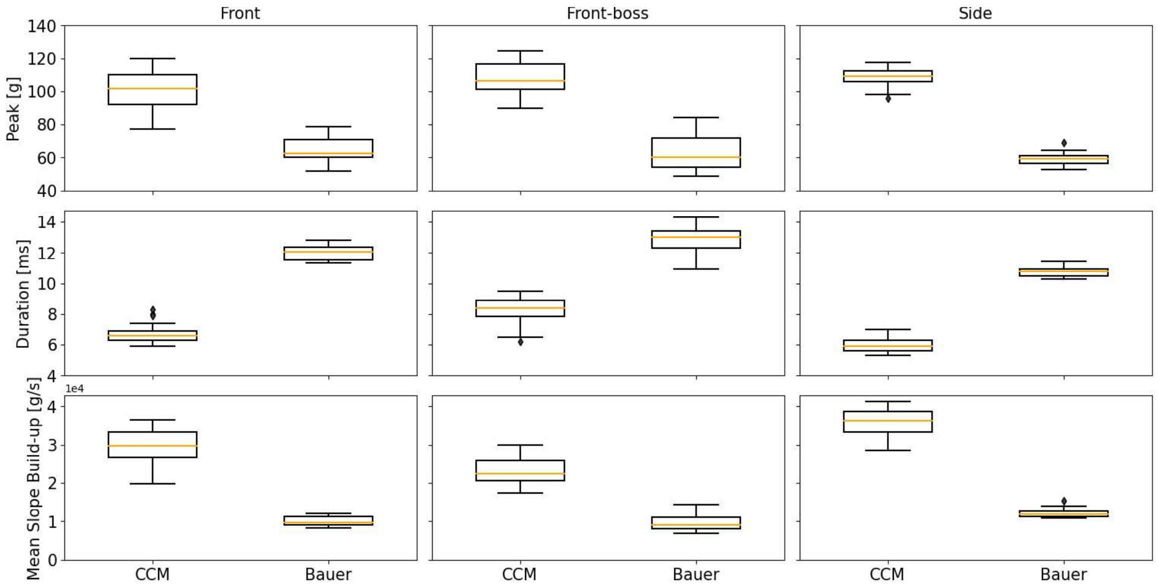

(b) 79 J

Figure s7: Comparison between CCM Tacks 910 and Bauer RE-AKT 150 helmets. The features were calculated on the main impact direction from the headform IMU and averaged across the impacts for each location. The mean slope was calculated on the raising part of the curves.
